# Supplementary material for: Spatio–temporal modelling of in vitro influenza A virus infection: the impact of defective interfering particles on type I interferon response
Source: bioRxiv. 2025 Oct 10:2025.10.09.681519. Preprint. [Version 1] doi: 10.1101/2025.10.09.681519 (PMC12632340; doi:10.1101/2025.10.09.681519)
Supplement: Supplementary file 1 [file NIHPP2025.10.09.681519v1-supplement-1.pdf]

bioRxiv preprint doi: https://doi.org/10.1101/2025.10.09.681519; this version posted October 10, 2025. The copyright holder for this preprint (which was not certified by peer review) is the author/funder, who has granted bioRxiv a license to display the preprint in perpetuity. It is made available under aCC-BY 4.0 International license.

Supporting Information

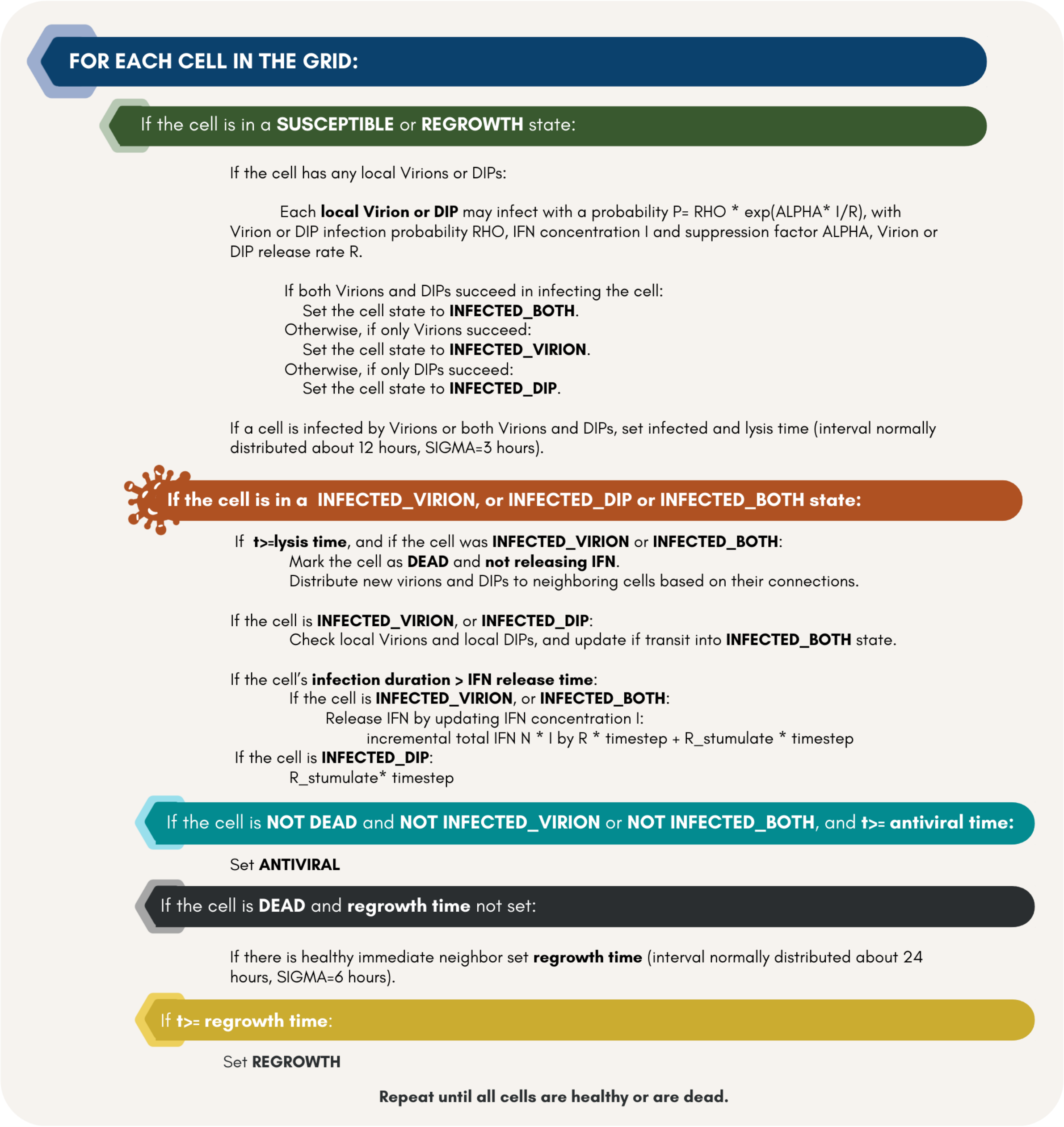

Figure S1: Model execution flowchart and explicit transition rules. The simulation begins by setting up a hexagonal grid where all cells are initially susceptible. Viruses and DIPs are introduced based on the inoculum size. Infection dynamics unfold through stochastic transitions: susceptible and regrowth cells become infected based on local particle and IFN levels. Infected cells undergo lysis, release particles and IFN, and die. Dead cells regrow if healthy neighbours are present. The model continues until termination criteria are met. Output includes time–resolved data and spatial maps. Animations available at: `https://shiny-spatial-infection-app-production.up.railway.app/`.

bioRxiv preprint doi: https://doi.org/10.1101/2025.10.09.681519; this version posted October 10, 2025. The copyright holder for this preprint (which was not certified by peer review) is the author/funder, who has granted bioRxiv a license to display the preprint in perpetuity. It is made available under aCC-BY 4.0 International license.

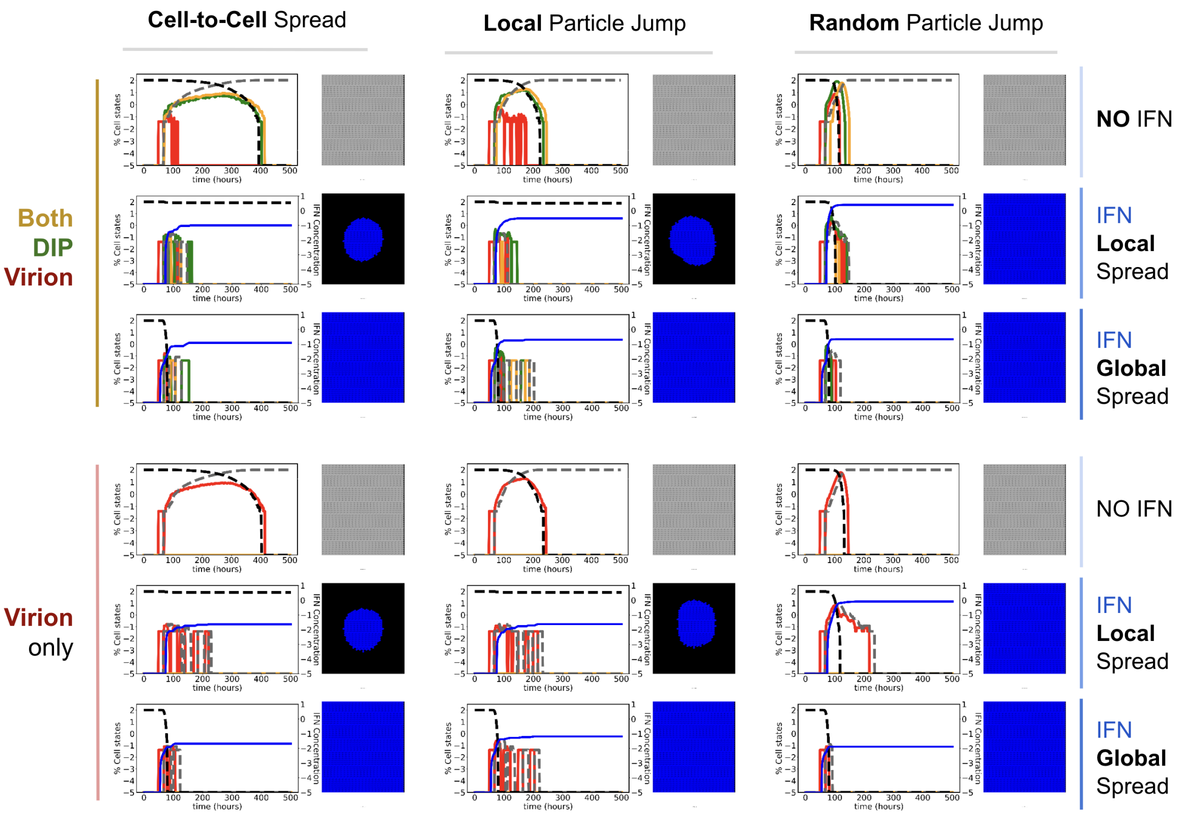

Figure S2: Dynamics of spread under varying particle–jump behaviours and IFN conditions. Outcomes are shown for scenarios with both DIPs and viruses (top three rows) and viruses only (bottom three rows), under three movement modes: cell–to–cell (left column), finite–radius jumps (middle column), and random jumps (right column). Each row corresponds to a different IFN mode: no IFN, finite–range IFN spread, and global/instantaneous IFN spread. **Left plot**: temporal dynamics. Both y–axes use a $\log_{10}$ scale: the left y–axis shows the percentage of cells in each state—**virus–only infected**, **DIP–only infected**, **co–infected**, **regrowth**, and **dead**; the right y–axis shows the **global IFN concentration** per cell. **Right plot**: the final spatial distribution of states: **virus–only**, **DIP–only**, **co–infected**, **antiviral**, **susceptible**, and **dead**.

bioRxiv preprint doi: https://doi.org/10.1101/2025.10.09.681519; this version posted October 10, 2025. The copyright holder for this preprint (which was not certified by peer review) is the author/funder, who has granted bioRxiv a license to display the preprint in perpetuity. It is made available under aCC-BY 4.0 International license.

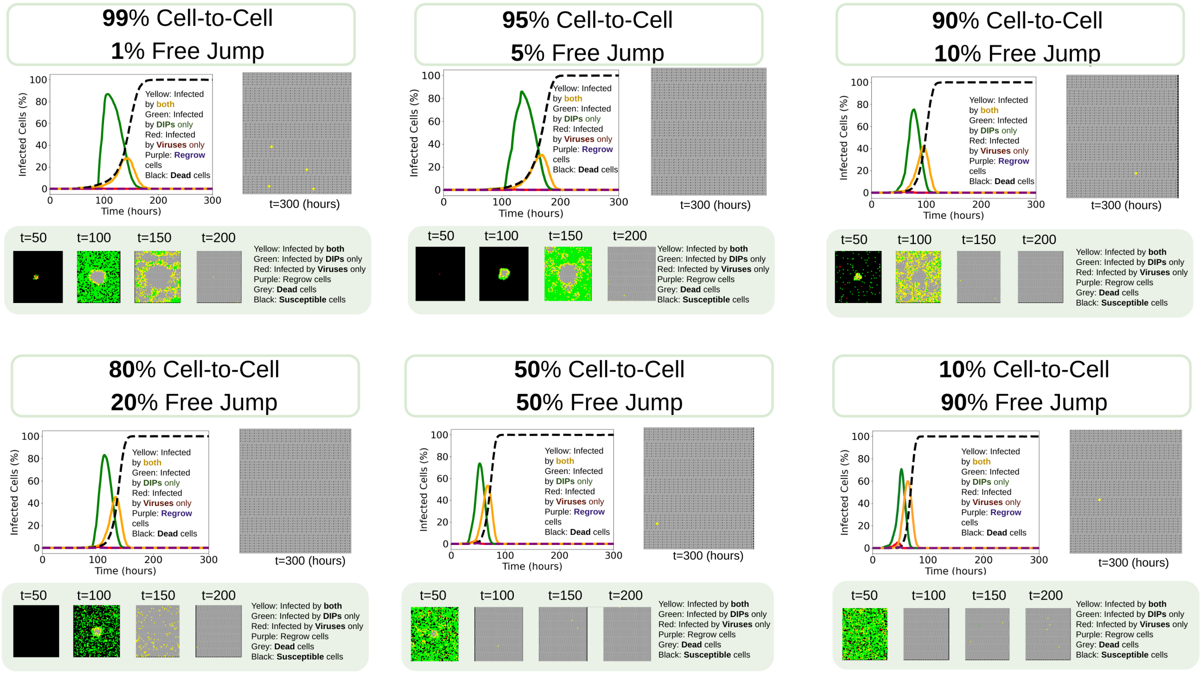

Figure S3: Heterogeneity in infection dynamics and plaque morphology under varying proportions of cell–to–cell and free–jump spread in Vero cells. Left panels: The line plots illustrate the percentage of infected cells over time, including viruses–only infections (red), DIPs–only infections (green), coinfections by both (yellow), regrowth cells (purple), and the dashed line represents dead cells (black). Right panels: The final plaque morphology at the simulation endpoint (t = 300 hours), highlighting the spatial distribution of cell states. At this time point, the plaques (grey) have nearly reached full coverage. Bottom panels: Plaque development over time at t = 50, 100, 150, and 200 hours, showing the progression of infection. Colours in the right and bottom panels represent different cell states: infected by both DIPs and viruses (yellow), infected by DIPs only (green), infected by viruses only (red), antiviral state (blue), dead cells (grey), uninfected/susceptible cells (black), and regrowth cells (purple).

bioRxiv preprint doi: https://doi.org/10.1101/2025.10.09.681519; this version posted October 10, 2025. The copyright holder for this preprint (which was not certified by peer review) is the author/funder, who has granted bioRxiv a license to display the preprint in perpetuity. It is made available under aCC-BY 4.0 International license.

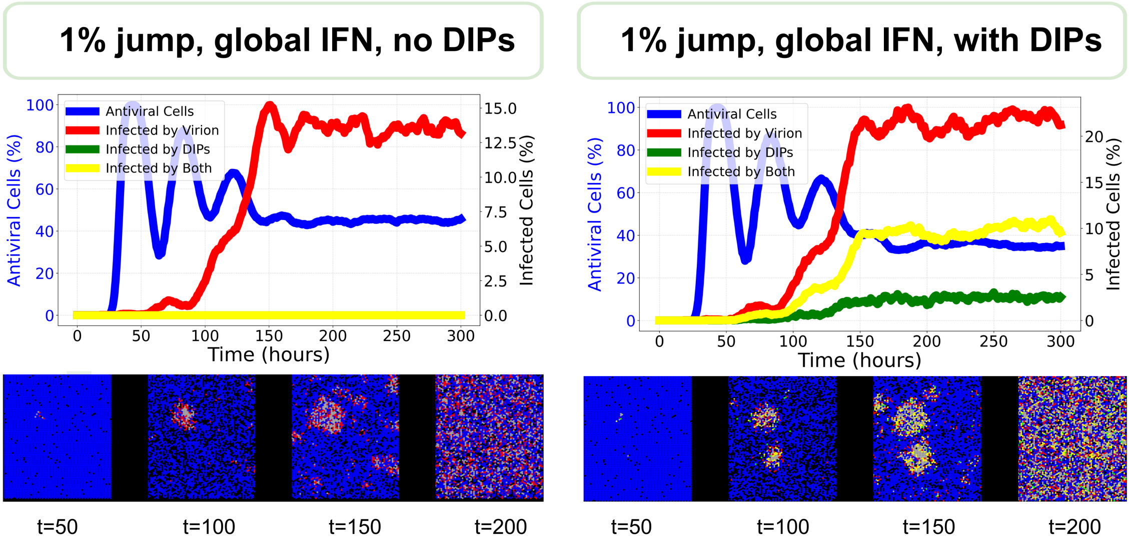

Figure S4: Comparison of plaque dynamics under global IFN with 1% free–jump transmission, in the absence (left) and presence (right) of DIPs. Top panels: time courses showing percentages of antiviral cells (blue, left y–axis), virus–infected cells (red), DIP–infected cells (green), and co–infected cells (yellow) (all right y–axis). Bottom panels: representative spatial snapshots at $t = 50, 100, 150,$ and 200 hours, illustrating how DIPs promote co–infection (yellow) and more diffuse plaque morphology despite similar levels of antiviral activation. Colour code: black, susceptible/uninfected; grey, dead; red, virus–infected; green, DIP–infected; yellow, co–infected; blue, antiviral.
